# A Channel Phase Error Correction Method Based on Joint Quality Function of GF-3 SAR Dual-Channel Images

**DOI:** 10.3390/s18093131

**Published:** 2018-09-17

**Authors:** Guangcai Sun, Jixiang Xiang, Mengdao Xing, Jun Yang, Liang Guo

**Affiliations:** 1 National Laboratory of Radar Signal Processing, Xidian University, Xi’an 710071, China; xjx.xjx@hotmail.com (J.X.); xmd@xidian.edu.cn (M.X.); 2 College of Geomatics, Xi’an University of Science and Technology, Xi’an 710000, China; yangjun_kx@163.com; 3 School of Physics and Optoelectronic Engineering, Xidian University, Xi’an 710071, China; lguo@mail.xidian.edu.cn

**Keywords:** dual-channel SAR, interchannel phase error, error correction, joint quality function, Heuristic search algorithm

## Abstract

Multichannel SAR is an effective approach to solving the contradiction between high azimuth resolution and wide swath. The goal of this paper is to obtain a new and effective method for estimating and compensating the interchannel phase error of the Chinese GF-3 Synthetic aperture radar (SAR). A channel phase error correction method based on the optimal value of the image domain quality function is proposed. In this method, the phase error is initially compensated using the correlation function method. In the fine correction of dual-channel phase error, a heuristic search algorithm is used to estimate the residual phase by searching the extremum of the quality function. After phase compensation in the image domain, the azimuth ambiguities caused by the remaining phase are eliminated. The proposed image domain processing method provides a new idea for channel phase error correction. The measured data of high-resolution GF-3 dual-channel ultrafine imaging mode verifies the validity of this method.

## 1. Introduction

Synthetic aperture radar (SAR) [1] is a high-resolution imaging radar that can use a wide-band signal and a long synthesis array to obtain a clear two-dimensional image of the observed area. Due to its high space orbit, the space-borne SAR platform is not affected by ground weather and national borders. It also has the advantages of large coverage [2,3], remote monitoring of moving targets [4], and strong antidestruction capability [5]. Based on the above advantages of space-borne SAR, space-borne SAR has become a hot spot for research and development in various countries. The Chinese GF-3, launched in 2016, is a C-band, multipolarized, high-resolution, remote-sensing satellite that can operate in 12 modes, and has a resolution up to 1 meter [6]. The high-resolution satellite’s imaging modes include traditional striping mode and scanning imaging mode, as well as wave imaging mode for ocean applications and global imaging mode [7,8,9]. GF-3 is a high-performance sensor, thus the processing of GF-3 data has become a hot topic in recent research.

Due to the increasing demand for applications in ocean observation, geological exploration, and environmental protection, the requirements for high-resolution and wide-swath (HRWS) [2,3,10] have been proposed for SAR imaging. In the HRWS SAR imaging process, in order to obtain a high azimuth resolution, a smaller antenna length needs to be set, and a high PRF (pulse repetition frequency) is required to solve the Doppler ambiguities. However, the high PRF results in range ambiguity and cannot obtain a wide swath [11]. Azimuth multichannel SAR can effectively solve this problem and realize HRWS. The Chinese GF-3 with two azimuth channels is one of the multichannel SARs. Because the PRF of the multichannel SAR system is lower than the Doppler bandwidth of the echo received by the receiving channel, the received echo of the system has Doppler ambiguity [12]. Multichannel SAR solves Doppler ambiguity through spectral reconstruction [13]. However, in the Doppler reconstruction of measured data, due to the existence of amplitude and phase errors between channels, the Doppler spectrum cannot be accurately reconstructed, which seriously affects the results of subsequent imaging processing [14]. Therefore, it is necessary to correct the phase errors of the GF-3 data before spectrum reconstruction in the imaging process.

The available channel error correction methods are mainly divided into two types: one is based on time domain, and the other is based on frequency domain. Some researchers have proposed a time domain channel error correction method based on the correlation characteristics of adjacent channels. Reference [15] explains that the interferometric phase of two adjacent channels consists of the Doppler centroid information and the difference of the phase biases between the adjacent channels, which can estimate the phase biases and the Doppler centroid simultaneously. To deal with the problems of a mismatch in channel phase and range sampling time, a method based on the azimuth cross-correlation is presented in [16]. With this type of method, due to the complexity of amplitude and phase errors between channels, a better image cannot be obtained by uniform compensation. Since the number of channels and the number of ambiguities are the same in the Chinese GF-3 dual-channel mode, a good SAR image without azimuth ambiguities is hard to obtain by using conventional processing methods. The frequency domain and time domain correction methods are represented by subspace-based correction methods (signal subspace and noise subspace) [3,17]. Reference [18] proposed a channel-calibration algorithm for the multichannel in azimuth airborne system, which is based on signal subspace (SSP) and has been adopted for the traditional SAR imaging. However, this method only considers the range-invariant channel error and it cannot be applied to HRWS SAR imaging with Doppler ambiguity. Moreover, a channel error calibration method for multichannel systems is studied in [19,20], assuming that Doppler ambiguity is free. For the distributed small satellite SAR system, a clutter-based channel error calibration algorithm was presented by [17], which applies active correction and self-correction techniques in array signal processing to channel error correction for multichannel in azimuth HRWS SAR system. The SSP method, which can calibrate the channel error, requires the channel number of the multichannel SAR to be greater than the Doppler ambiguities number, so that the signal subspace and the noise subspace can be constructed to conduct channel phase error estimation [21]. However, in GF-3 SAR, the number of channels is equal to the Doppler ambiguities number, and thus the noise subspace cannot be accurately constructed. In this case, the amplitude and phase errors between the two channels cannot be accurately estimated. In the case where noise subspace is unable to be constructed in GF-3, reference [22] proposed a weighted minimum entropy channel error correction method, which uses image entropy as an indicator to estimate the channel phase.

For the problem faced above, a channel phase error correction method based on joint quality function of GF-3 SAR dual-channel images is proposed in this paper. In coarse correction, interchannel error correction is performed using a correlation function. Due to the complexity of phase error between channels, the channel still has residual phase. A joint quality function is defined in the image domain to evaluate the degree of azimuth ambiguities of the image for the precious phase correction. The heuristic search algorithm finds the optimal value of the joint quality function to estimate the residual phase of the channel. The signal azimuth spectrum reconstruction is completed after phase compensation in the image domain. Compared with the conventional channel phase error estimation method, the proposed method has a wider application scenario and does not require extra spatial degrees of freedom for error estimation. With the number of channels determined, more imaging data with ambiguities numbers can be processed than conventional channel error estimation methods. More importantly, since the phase error correction is based on the image domain compared to the estimation method in the frequency domain in the reference, the proposed method directly relates the interchannel phase error to the ambiguities of the SAR image. Compared with the method in [22], the proposed method in this paper has a small amount of computing as the result of imaging processing once before the iteration. Therefore, this method is more intuitive and efficient.

This paper starts with a brief introduction of the dual-channel SAR model in Section 2. In Section 3.1, rough corrections based on correlation functions are introduced. In Section 3.2, a phase error correction method is provided. The definition of the quality function is first proposed in Section 3.2.1, and then the residual phase search method based on the heuristic search algorithm is introduced in Section 3.2.2. At the end of Section 3.2, the flowchart of the precision correction algorithm is given in Section 3.2.3. In Section 4, we use the method proposed in this paper to process the measured data of GF-3. The results of the analysis verify the effectiveness of the proposed method. The last section is a summary of the method and prospects for future application scenarios.

## 2. Dual-Channel SAR Model

In a single-channel SAR system, the azimuth high resolution and wide swath are a set of contradictions [23]. This set of contradictions is essentially a contradiction between large azimuth bandwidth and low sampling frequency. Using the azimuth multichannel method, each channel echo is the result of delayed sampling of the previous channel echo [24], which is equivalent to having a higher azimuthal sampling rate. The GF-3 is a dual-channel ocean observation satellite. This article focuses on the dual-channel model of GF-3. In this paper, it is considered that the GF-3 satisfies the following equation:

(1) v/PRF=Md

where *v* is the velocity of space-borne platforms, PRF is pulse repetition frequency, *M* is the number of receiving channels, *d* is the distance between the equivalent phase centers. The phase center distribution satisfying the above conditions is called DPC (Displaced Phase Center) [13]. After processing by the equivalent phase center, it can be considered that each channel of the dual-channel SAR system independently transmits and receives signals synchronously in the center of the equivalent phase. It is considered herein that the GF-3 model strictly satisfies the DPC conditions.

Let the time domain signals of channel 1 and channel 2 be $S_{1}\left(\hat{t},t_{m}\right)$ and $S_{2}\left(\hat{t},t_{m}\right)$, where $\hat{t}$ is the range fast time. In the 2-D time domain, the echo of the single channel in the center of the phase can be expressed as

(2) $$s_{1}\left(\hat{t},t_{m}\right)=a_{r}\left(\hat{t}-\frac{2R\left(t_{m}\right)}{c}\right)a_{a}\left(t_{m}\right)\exp\left[j\pi \gamma {\left(\hat{t}-\frac{2R\left(t_{m}\right)}{c}\right)}^{2}\right]\exp\left[-j\frac{4\pi }{\lambda }R\left(t_{m}\right)\right]$$

and

(3) $$\begin{array}{cc} s_{2}\left(\hat{t},t_{m}\right) & =S_{1}\left(\hat{t},t_{m}-\frac{d}{v}\right)\\ & =a_{r}\left(\hat{t}-\frac{2R\left(t_{m}\right)}{c}\right)a_{a}\left(t_{m}-\frac{d}{v}\right)\times \exp\left[j\pi \gamma {\left(\hat{t}-\frac{2R\left(t_{m}-d/v\right)}{c}\right)}^{2}\right]\exp\left[-j\frac{4\pi }{\lambda }R\left(t_{m}-\frac{d}{v}\right)\right] \end{array}$$

where λ is the wave length, $t_{m}$ is the azimuth slow time, $\hat{t}$ is the range fast time, and $R\left(t_{m}\right)=\sqrt{R_{B}^{2}+{\left(vt_{m}\right)}^{2}}$, where $R_{B}$ is the distance of closest approach, γ is the chirp rate, and $a_{r}\left(\cdot \right)$ and $a_{a}\left(\cdot \right)$ are the window function in range and azimuth, respectively.

After the echo data is in the range pulse compression, the range signal of two channels expressed by discrete form in the time domain is

(4) $$s_{1}\left(m,t_{m}\right)=s_{1}\left(\hat{t},t_{m}\right)$$

(5) $$s_{2}\left(m,t_{m}\right)=s_{2}\left(\hat{t},t_{m}\right)$$

**Figure 1:**
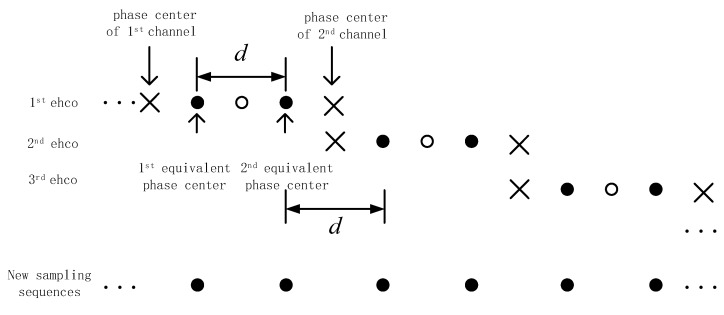
Increasing of azimuth sampling by multiphase center.

It can be seen from the formula that the echo signal of Channel 2 is a delay of the echo signal of Channel 1 and can be obtained by discrete sampling of the formula.

(6) $$s_{1}\left(m,n\right)=s_{1}\left(m,t_{m}=\frac{n}{PRF}\right)$$

(7) $$\begin{array}{cc} s_{2}\left(m,n\right) & =s_{2}\left(m,t=\frac{n}{PRF}\right)\\ & =s_{1}\left(m,\frac{n}{PRF}-\frac{d}{v}\right)\\ & =s_{1}\left(m,\frac{n-1/2}{PRF}\right) \end{array}$$

where n=1,2,⋯,N, *N* is the number of single-channel data azimuth sampling points. The azimuth ambiguities exist in the discrete azimuth signals of each channel. Insert zero between every two sample points of 1 and 2. The two channels of discrete data after interpolation are expressed in the following form

(8) $$T_{1}\left(m,\xi \right)=\left\{\begin{array}{c} s_{1}\left(m,\xi /2\right),\xi =2n\\ 0,\xi =2n-1 \end{array}\right\}$$

(9) $$T_{2}\left(m,\xi \right)=\{\begin{array}{cc} 0 & ,\xi =2n\\ s_{2}\left[m,\left(\xi +1\right)/2\right] & ,\xi =2n-1 \end{array}$$

where n=1,2,⋯,N. Because the DPC conditions are strictly met, the dual-channel nonambiguous signals can be obtained by direct rearrangement. Two-channel data rearrangement is expressed as

(10) $$s\left(m,\xi \right)=T_{1}\left(m,\xi \right)+T_{2}\left(m,\xi \right)$$

where ξ=1,2,⋯,2N. The odd-numbered position sampling point of s(m,ξ) represents the echo of Channel 1, and the even-numbered position represents the echo of Channel 2. The PRF of the rearranged dual channel azimuth signal sample sequence is doubled with respect to the single channel signal. The above process can be expressed as the following schematic in Figure 1:

Let the phase error between the two channels be $\theta_{0}$, and the discrete azimuth signal with error is expressed as

(11) $$s_{e}\left(m,\xi \right)=T_{1}\left(m,\xi \right)+\exp\left(j\theta_{0}\right)T_{2}\left(m,\xi \right),\xi =1,2,\cdots ,2N$$

The processing of the azimuth signal by the imaging algorithm is indicated by *H*, and the azimuth information of the azimuth signal is represented as *I*. It can be considered that *H* is a linear transformation, then imaging process of $s_{e}$ is expressed as the following formula.

(12) $$\begin{array}{cc} I_{e}\left(m,\xi ;\theta_{0}\right) & =H\left[s_{e}\left(m,\xi \right)\right]\\ & =H\left[T_{1}\left(m,\xi \right)+\exp\left(j\theta_{0}\right)T_{2}\left(m,\xi \right)\right]\\ & =H\left[T_{1}\left(m,\xi \right)\right]+H\left[T_{2}\left(m,\xi \right)\exp\left(j\theta_{0}\right)\right]\\ & =I_{1}\left(m,\xi \right)+I_{2}\left(m,\xi \right)\exp\left(j\theta_{0}\right) \end{array}$$

where $I_{1}\left(m,\xi \right)$ and $I_{2}\left(m,\xi \right)$ indicate the azimuth information of the respective imaging results of the two channel echoes, respectively. Due to the presence of phase error between channels, the DPC condition is no longer strictly met. Therefore, after the two-channel data are respectively imaged using chirp scaling algorithm (CSA) [25] and data is rearranged after zero insertion, the azimuth ambiguities will still exist on the image. To realize the suppression of the azimuth ambiguities in the image, it is necessary to estimate and compensate the interchannel phase error.

## 3. Dual-Channel Data Phase Error Correction Method

This section may be divided by subheadings. It should provide a concise and precise description of the experimental results and their interpretation, as well as the experimental conclusions that can be drawn.

### 3.1. Phase Error Coarse Correction

For the phase error of dual-channel signals, the correlation function method is used to perform coarse correction of phase error. The cross-correlation function of the radar echoes received by these two channels is as follows:

(13) $$\begin{array}{cc} C_{2,1} & =E\left[s_{2}\left(\hat{t},t_{m}\right)\cdot s_{1}^{H}\left(\hat{t},t_{m}\right)\right]\\ & ={A_{1}}^{2}\cdot \exp\left(j\left(\phi_{c,2}-\phi_{c,1}\right)\right) \end{array}$$

where E(⋅) denotes the statistically expected operation and ${\left(\cdot \right)}^{H}$ denotes the conjugation operation. Because it works in the side-looking mode, the above equation can get the phase error between the two channels as

(14) $$\phi =\phi_{c,2}-\phi_{c,1}=∠\left(C_{2,1}\right)$$

where $\phi_{c,1}$ and $\phi_{c,2}$ are the phase of the echo of the two channels, respectively, and ∠(⋅) denotes the angular operator. In the above analysis, the SAR echoes interfere in the two-dimensional time domain, that is, conjugate multiplication. After the relative phase error ϕ is obtained, phase compensation is performed using ϕ for the echo of channel 2, so that the phase error coarse correction of the dual-channel SAR can be completed.

The correlation function-based channel error correction method discussed above is discussed in the case where the squint angle is zero. In general, the squint angle is not equal to zero. In this case, reference [16] proposes a channel phase error estimation method that combines Doppler centroid estimation.

**Figure 2:**
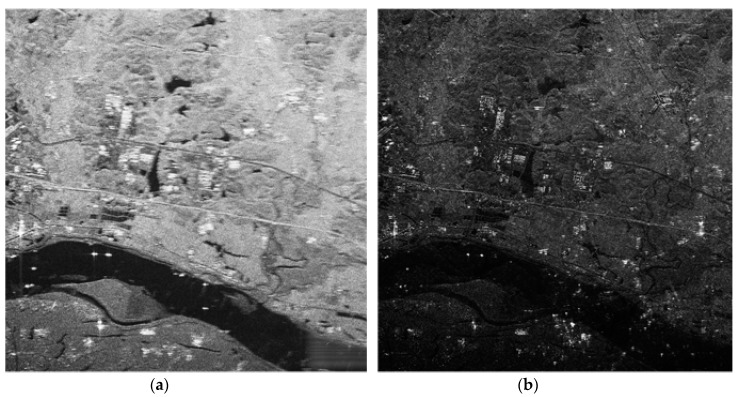
Reconstruction signal imaging results before and after interference phase compensation. (**a**) Imaging result after direct reconstruction. (**b**) Imaging result after interference phase compensation.

After phase error coarse correction, residual phase error between channels θ is $\left|\theta_{0}-\phi \right|$. The method in Section 2 is used to rearrange the double-channel data and obtain the signal after direct reconstruction. CS imaging was performed on the reconstructed signal. Figure 2 shows reconstruction signal imaging results before and after rough compensation, where Figure 2a shows direct reconstruction imaging results. The result of the imaging scene is messy and fuzzy, and the image is not clear. This situation is particularly obvious in the upper left corner town area where the ground scene is more complex. Figure 2b shows the reconstruction results after rough compensation, and the ambiguous components are mostly suppressed. The imaging scene in the upper right town area is clearly visible, but there are still some ambiguities in the lower part of the picture.

### 3.2. Phase Error Precision Correction

In the rough correction process in Section 3.2, the phase error is coarsely compensated using the interchannel interference phase information. However, when the Doppler spectrum is ambiguous, channel phase error cannot be accurately estimated by correlation function method. In order to further suppress the ambiguous component and improve the image quality, the remaining phase of the channel needs to be accurately estimated. In this section, an optimal phase error correction method based on the joint quality function of the image domain is proposed.

#### 3.2.1. Joint Quality Function Definition

In Section 2, the expression of the azimuth information of the SAR image without ambiguities can be obtained when θ is equal to zero;

(15) $$I\left(m,\xi \right)=I_{1}\left(m,\xi \right)+I_{2}\left(m,\xi \right)$$

Then

(16) $$\begin{array}{cc} \Delta & =I_{e}\left(m,\xi ;\theta \right)-I\left(m,\xi \right)\\ & =\left[I_{1}\left(m,\xi \right)+I_{2}\left(m,\xi \right)\exp\left(j\theta \right)\right]-\left[I_{1}\left(m,\xi \right)+I_{2}\left(m,\xi \right)\right]\\ & =I_{2}\left(m,\xi \right)\left[1-\exp\left(j\theta \right)\right] \end{array}$$

where Δ indicates the effect form of interchannel phase error on the SAR image. If θ is a small phase, the relationship between θ and Δ approximately satisfies the relationship of the quadratic function. Obviously, if θ is accurately estimated and compensated, Δ is zero, and ambiguities are depressed completely. However, I(m,ξ) is not possible to get in practice.

Based on the analysis above, in order to reflect the gradual improvement trend of image ambiguities in the process of residual phase error precision estimation, this paper defines the joint quality function according to the square of the amplitude of the ambiguous region. The expression of the joint quality function is as follows.

(17) $$C_{amb}\left(\varphi \right)=\sum_{\xi =1}^{N}\sum_{m=1}^{M}\frac{{\left|I_{e}\left(m,\xi ;\theta \right)\right|}^{2}-{\left|I_{e}\left(m,\xi ;\varphi \right)\right|}^{2}}{{\left|I_{e}\left(m,\xi ;\theta \right)\right|}^{2}}$$

where φ is the phase of precision correction, M and N are the number of sample points in the range and azimuth of the image, respectively. $I_{e}\left(m,\xi ;\theta \right)$ is the 2-D discrete expression of dual-channel data with phase error θ, and presents the ambiguous SAR image obtained after signal reconstruction in the case that the phase error is unknown. $I_{e}\left(m,\xi ;\varphi \right)$ indicates the SAR image after the phase φ is compensated to Channel 2 and the signal is reconstructed. If θ is equal to 0, $C_{amb}\left(\varphi \right)$ is 0, which indicates $I_{e}\left(m,\xi ;\theta \right)$ is equal to I(m,ξ). If φ equals θ, $I_{e}\left(m,\xi ;\varphi \right)$ is denoted that the phase error of Channel 2 is accurately compensated and a SAR image without ambiguities can be obtained. In this case, ${\left|I_{e}\left(m,\xi ;\theta \right)\right|}^{2}-{\left|I_{e}\left(m,\xi ;\varphi \right)\right|}^{2}$ represents the power of ambiguities and the value reaches its maximum value. Joint quality function indicates the degree of suppression of the ambiguities component after the phase φ is compensated. The joint quality function directly establishes the mathematical model between the residual phase error and the degree of azimuth ambiguities of the image.

#### 3.2.2. Heuristic Phase Search Algorithm

According to the previous analysis, when using the joint quality function as a criterion for evaluating the situation of the phase error correction, it is desirable to find a phase to maximize the value of the joint quality function as follows:

(18) $$\varphi_{m}=\underset{\varphi }{\arg\max}\left(C_{amb}\left(\varphi \right)\right)$$

where $\varphi_{m}$ is the maximum of $C_{amb}\left(\varphi \right)$.

This paper introduces a method of hill-climbing algorithm (HCA) [26,27], which is a kind of heuristic searching algorithm. This method extends the current node and evaluates its child in the search process, and the optimal child is selected and further extended. When a search reaches a state that is better than all of its children, the search stops. HCA-based heuristic phase error search algorithm allows the following steps to be included in the processing:

- Take 1 as the current phase search node.
- A set of phases is formed by extending to both sides in a certain step with the current node as a center.
- Calculate the value of the joint quality function corresponding to this set of phases.
- The phase that maximizes the joint quality function will be the current node of the next iteration.
- Repeat steps 2–5 until a termination condition is met.

**Figure 3:**
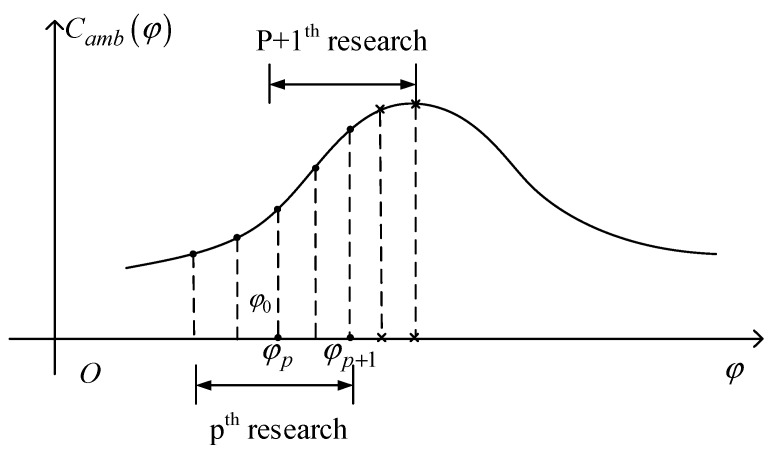
Process of heuristic phase error search algorithm.

Since the interchannel phase error after coarse correction can be considered to be very small, it can be considered in this case that the relationship between φ and $C_{amb}\left(\varphi \right)$ approximately satisfies the relationship of the quadratic function as well. This approximation was verified in the measured data processing. A schematic diagram of a heuristic phase search algorithm is shown in Figure 3:

In Figure 3, $\varphi_{p}$ is the current node of the *p*th search. According to step $\varphi_{0}$, take two points on the left and right sides of the point and get a total of five points. Calculate the joint quality function values of these five points, and use the node with the highest joint quality function value as the current node of the (*p* + 1)th search. By iterating this operation, the final phase error estimated will converge to the actual residual phase error between the channels. All nodes of the *p*th search can be represented as the following vectors:

(19) $$L_{p}=\left[\begin{array}{ccccc} \varphi_{p}-2\varphi_{0} & \varphi_{p}-\varphi_{0} & \varphi_{p} & \varphi_{p}+\varphi_{0} & \varphi_{p}+2\varphi_{0} \end{array}\right]$$

where $\varphi_{p}$ denotes the phase of compensation in the *p*th search, and $\varphi_{0}$ is the length of step in search and can be smaller with the search process.

Iterative operations usually end up satisfying the constraints. The joint quality function threshold is set to determine if the search should be stopped. If the search joint quality function value is no longer significantly raised in the two adjacent searches, the search is stopped, and it is considered that searching for the optimal solution is performed in this search. Set the constraints as follows:

(20) $$\Delta C_{amb}\left(\varphi_{p+1},\varphi_{p}\right)=C_{amb}\left(\varphi_{p+1}\right)-C_{amb}\left(\varphi_{p}\right)$$

(21) $$\left|\Delta C_{amb}\left(\varphi_{p+1},\varphi_{p}\right)\right|\leq \varepsilon_{0}$$

where $\left|\Delta C_{amb}\left(\varphi_{p+1},\varphi_{p}\right)\right|$ indicates the amount of change in the joint quality function in two consecutive searches, $\varepsilon_{0}$ is the threshold. The smaller the $\varepsilon_{0}$ is, the less the suppression of the ambiguities increases, the higher the error estimation accuracy between the channels, and the higher the amount of computation. Therefore, it is usually necessary to consider the compromise between the amount of calculation and the accuracy of the error.

#### 3.2.3. Precision Correction Process

The definition of the joint quality function and the heuristic search algorithm is introduced in the first two subsections. Phase error precision correction is described in this section. The following figure shows the flow chart of this process.

**Figure 4:**
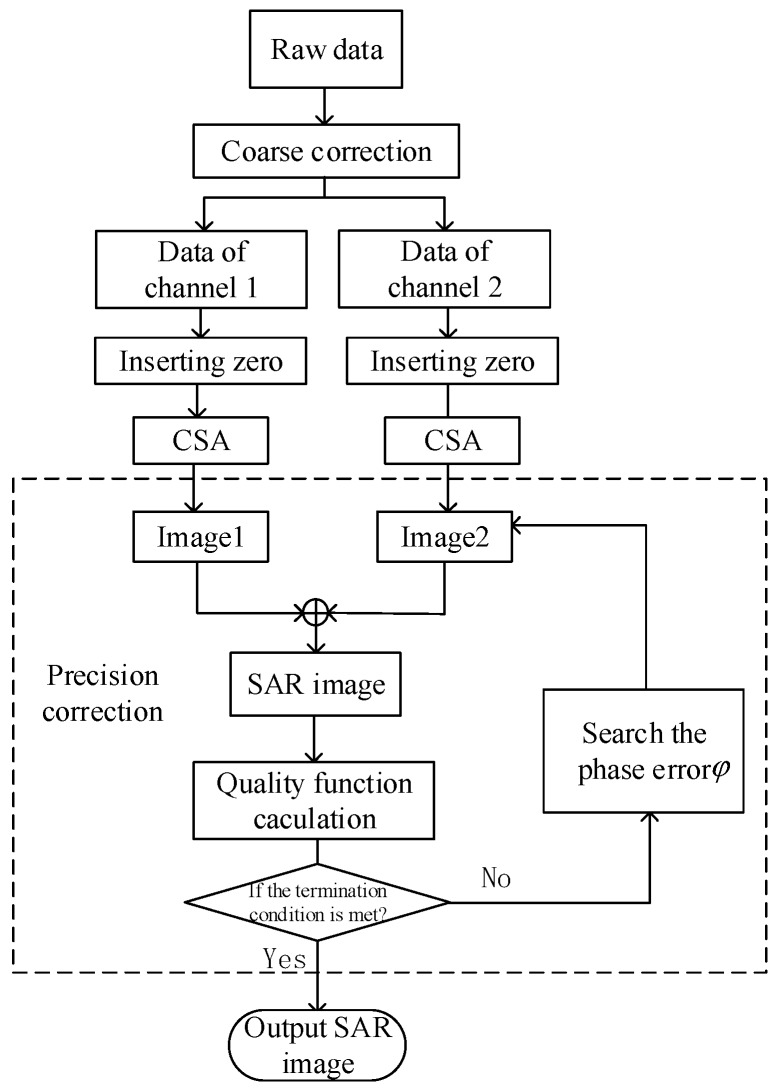
The work flow of the precision correction.

As shown in Figure 4, coarse correction is performed on the raw data firstly. Then, according to the method of Section 2, insert two zeros for each channel. The data of two channels inserted after zeroing are respectively imaged with CSA. Due to the channel phase error, if the data of the two channels is directly rearranged, the image is ambiguous. Therefore, precise correction of the channel phase error is required. The phase is searched using the heuristic search algorithm in Section 3.2.3, and the phase in each search is compensated to Image2 in the image domain. The images of the two channels are rearranged according to the Formula (10) and their joint quality functions are calculated. Finally, according to Formula (20), it is judged whether the termination condition of Formula (21) is satisfied. If the termination condition is satisfied, the final image is output, otherwise the search continues.

## 4. Experiments

In precision correction, the iterative search algorithm is used to estimate the residual phase error through multiple iterations. The phase error is compensated in the image domain and the signal is reconstructed by data rearrangement. By this method, the azimuth ambiguities caused by the interchannel phase error can be eliminated and a higher quality SAR image can be obtained. The following shows the change of image ambiguities during the iteration.

**Figure 5:**
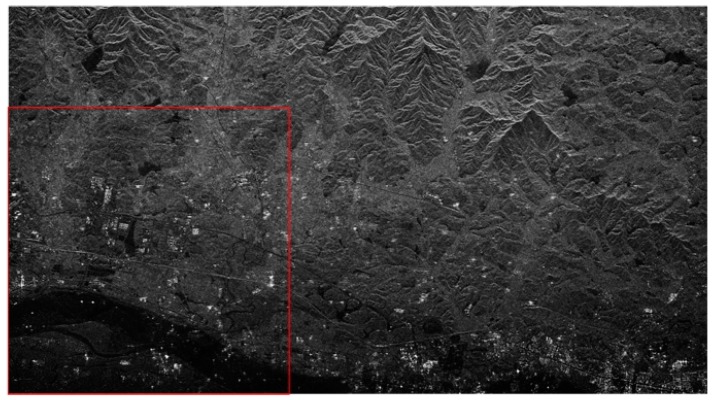
Reconstruction of imaging results after the coarse correction (the initial sample).

**Figure 6:**
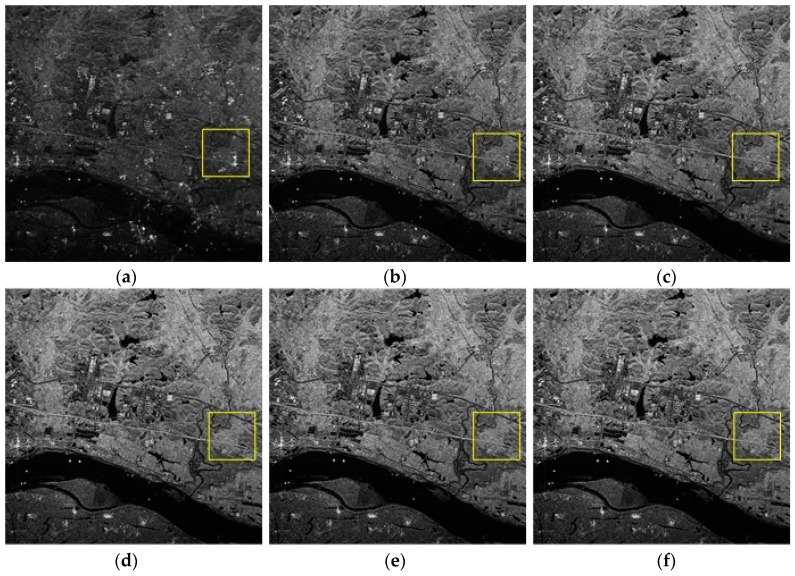
The ambiguous region in the red box of the initial sample: (**a**) the initial sample, (**b**) 1st iteration, (**c**) 2nd iteration, (**d**) 3rd iteration, (**e**) 4th iteration, (**f**) 5th iteration.

Figure 5 is the SAR image after first-step phase error correction. For simplicity, we select the region in the red box as the initial sample for iteration. Figure 6a shows the partial results of the correction in the first step, which is also the starting sample in the second step of the precision estimation process. During the iteration, select the yellow-box-covered region in Figure 6 to visually reflect the ambiguities suppression effect, and the corresponding number of two-dimensional sampling points is 600×600. The value of the joint quality function is calculated by substituting the amplitude of the ambiguous region after the compensation for each iteration into Equation (17). After several iterations to estimate the residual phase error, the signal is compensated and reconstructed. The final imaging results are shown in Figure 6b–f. From Figure 6a,f, it can be clearly seen that the ambiguities component gradually decreases and the image becomes more distinct after each estimation compensation. The ambiguities component appearing in the lake area due to the presence of the residual phase is gradually suppressed after each iteration for phase compensation. After five iterations, the image of the lake area in the final imaging result is very pure, and the ambiguities component has been well suppressed. By estimating the ambiguities suppression of the region in the yellow box, the ambiguities in the central region of the SAR image (Figure 5) is also suppressed.

In addition, in order to quantitatively describe the effect of improving the ambiguities suppression, the joint quality function and the amount of ambiguities suppression change for each calculation are recorded in Table 1.

As shown in Table 1, the ambiguities suppression variation for the last two iterations is 1.57%, which is less than the set threshold of 2%. At this time, the corresponding maximum joint quality function value reaches 88.75%, which satisfies the application requirements. Since there is inherent target information in the region of the sampling, the value of the joint quality function calculated by Equation (1) cannot reach 100%. In addition, the more complex the selected scene, the smaller the value of the calculated joint quality function.

Data processing is performed using the SSP method in [17] and the time domain correlation method in [15].

**Figure 7:**
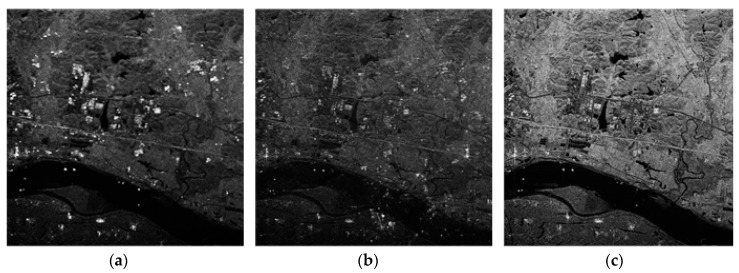
Comparison of the method in this paper with [15,17]. (**a**) The SSP method in [17]. (**b**) Time domain correlation method in [15]. (**c**) Proposed method in this paper.

Figure 7b is the result of the first step of the rough correction in this paper, and also the initial sample of the second-step correction. Due to the existence of the residual phase, Figure 7b has a fuzzy presence compared to the iterative result of Figure 7c. Figure 7a shows the result of processing using the signal subspace method. This method is to estimate the channel error by constructing the noise subspace in the frequency domain. However, the GF-3 dual-channel SAR has no extra spatial freedom to accurately estimate the channel phase error. Therefore, it can be seen from the figure that in this case, the treatment effect by this method is not good, and the suppression of the azimuth ambiguity cannot be achieved. Therefore, the proposed method has an obvious advantage in this case compared with the traditional method.

## 5. Discussion

This method realizes channel error compensation for GF-3 dual-channel SAR through two-step correction, including coarse correction and fine correction. Through the data processing of the Chinese GF-3 SAR system, the ambiguities component of the SAR image is gradually suppressed with the increase of the number of iterations, which verifies the effectiveness of the “coarse to fine” channel phase error correction method proposed in this paper. In the fine correction, a joint quality function is defined in the image domain to quantify the ambiguous degree of the image. This method of phase estimation and correction by evaluating the joint quality function of the image domain makes the image ambiguities directly related to the channel phase error and is simpler and more efficient than the available methods. Because the method of this paper operates in the image domain and only needs to image the data once, it is more practical and efficient in practical applications. More importantly, the method proposed does not need to perform channel error estimation through redundant spatial degrees of freedom. This determines that the application scenario of the method in actual operation is more extensive than the conventional channel error estimation method.

Future efforts include extending the method in this paper to the case with more channel in azimuth, and finding the joint quality function in time domain suitable for such cases. In addition, more complex error influencing factors should be taken into account to improve this method and enhance the ability to control ambiguity.

## Figures and Tables

**Table 1:** The record of joint quality function value with iteration.

	Initial Sampling	1st Search	2nd Search	3rd Search	4th Search	5th Search
Average power	1568	829.3	441.7	301.7	201.0	176.4
$C_{amb}\left(\varphi \right)$	0	47.11%	71.83	80.76%	87.18%	88.75%
$\left|\Delta C_{amb}\left(\varphi_{p+1},\varphi_{p}\right)\right|$	0	47.11%	24.72%	8.93%	6.42%	1.57%
